# *LhSBP1* Gene of *Liriodendron* Hybrid Enhances the Cold Resistance of Plants by Regulating ROS Metabolism

**DOI:** 10.3390/plants15020196

**Published:** 2026-01-08

**Authors:** Tian Min, Yinyue Zuo, Teja Manda, Yuchen Li, Ye Lu, Haibin Xu, Jinhui Chen, Liming Yang

**Affiliations:** 1 State Key Laboratory of Tree Genetics and Breeding, College of Life Sciences, Nanjing Forestry University, Nanjing 210037, China; tmin@njfu.edu.cn (T.M.); zuoyinyue@njfu.edu.cn (Y.Z.); teja.manda@njfu.edu.cn (T.M.); liyuchen@njfu.edu.cn (Y.L.); xuhaibin@njfu.edu.cn (H.X.); 2 College of Forestry, Nanjing Forestry University, Nanjing 210037, China; luye@njfu.edu.cn

**Keywords:** *LhSBP1*, cold stress, reactive oxygen species

## Abstract

Selenium-Binding Protein 1 (SBP1), involved in selenium metabolism, contributes to plant stress response. However, it is currently unknown whether the SBP1 protein from *Liriodendron* hybrid (LhSBP1) plays a role in response to cold stress. In this study, transgenic overexpression lines of *LhSBP1* in *Arabidopsis thaliana* and *Populus deltoides × P. euramericana* cv. ‘Nanlin 895’, were used as materials to conduct phenotypic observations and physiological and biochemical determinations under cold stress. The results showed that the full-length CDS sequence of *LhSBP1* gene was cloned, with a length of 1467 bp, encoding 488 amino acids. Under cold stress, physiological and biochemical indexes showed that the contents of reactive oxygen species (ROS) and malondialdehyde (MDA) in transgenic *Arabidopsis* were lower, with the contents of hydrogen peroxide (H_2_O_2_) and superoxide anion (O_2_^−^) being 0.72 and 0.71 times those of the wild type, respectively, and the MDA content was 0.53 times that of the wild type. Compared with the wild type, the activities of antioxidant enzymes including superoxide dismutase (SOD), peroxidase (POD) and catalase (CAT) increased by 1.2, 1.75, and 1.48 times respectively, and the soluble protein content increased by 1.41 times, which significantly improved the cold tolerance of *Arabidopsis*. The contents of H_2_O_2_, O_2_^−^, and MDA in *LhSBP1* transgenic ‘Nanlin 895’ poplar were 0.63 and 0.67 times and 0.6 times those of wild type, respectively. The activities of SOD, POD and CAT were increased by 1.37, 1.48, and 1.44 times, and the soluble protein was increased by 1.28 times, which significantly improved the cold tolerance of ‘Nanlin 895’ poplar. Taken together, this study utilized two model plant systems to demonstrate the positive and conserved functions of *LhSBP1* in plant cold tolerance defense response, which provided valuable genetic resources for the breeding of cold-tolerance woody plants.

## 1. Introduction

During the growth and development of plants, they are exposed to various environmental stresses [1], which can be specifically classified into biotic stresses and abiotic stresses [2]. Among these, abiotic stresses mainly include heat stress, cold stress, drought stress, salt stress, and heavy metal pollution [3]. In recent years, global climate warming has intensified, leading to an increase in extreme weather events. Low temperature is one of the major environmental factors limiting plant growth, productivity, and geographical distribution. When exposed to low temperatures, plant cells undergo a variety of physiological and biochemical reactions, including the accumulation of osmoprotectants and protective proteins such as soluble sugars, soluble proteins, proline, and cold-responsive proteins [4]. These proteins contribute to maintaining cell membrane integrity and scavenge reactive oxygen species (ROS), which are essential for plant adaptation to cold stress [5]. Related research suggests that plant responses to cold stress are closely related to ROS signaling. Under cold conditions, plants often increase the activity of antioxidant enzymes such as peroxidase (POD), superoxide dismutase (SOD), and catalase (CAT) to scavenge excess ROS and reduce oxidative damage [6]. ROS play a dual role in plants: at moderate levels, they act as signaling molecules that regulate plant growth and development, thereby promoting adaptability to adverse circumstances, whereas excessive accumulation causes cellular damage. Therefore, maintaining cellular ROS equilibrium is critical for plant survival [7]. Lipids and their derivatives are fundamental parts of living organisms, and malondialdehyde (MDA), a product of lipid peroxidation, is widely used as an indicator of oxidative stress and membrane damage [8].

To cope with cold stress, plants have evolved complex and sophisticated molecular regulatory networks to maintain cellular homeostasis and enhance stress tolerance [9]. Low temperatures can stimulate receptors located on the cell membrane. The low-temperature signal induces gene expression through multiple pathways, leading to the production of corresponding proteins that enhance plants’ cold tolerance [10]. At present, the most thoroughly studied low-temperature response signaling pathway in plants is the ICE1-CBF-COR cold response pathway, which is recognized as the primary pathway conferring cold tolerance in plants. Many cold acclimation-related genes are located downstream of CBF [11,12]. CBF/DREB1 is an important transcription factor regulating plant growth and development, as well as plant responses to cold stress [13]. Its encoded protein binds to the DRE/CRT cis-acting element in the promoter region of downstream genes, activating the expression of cold stress-responsive genes such as CORs, thereby improving the cold tolerance of plants [14]. Similarly, MYB and WRKY transcription factors (TFs) play crucial roles in integrating cold stress signals and regulating antioxidant defense systems [15,16]. *FvMYB44* from *Fragaria vesca* enhances cold and salt tolerance by modulating antioxidant enzyme activity [17], while *MbWRKY50* from *Malus baccata* improves cold and drought resistance by activating downstream stress-responsive genes [18]. Additionally, NAC and bZIP transcription factors have been shown to participate in the crosstalk of cold stress responses, further expanding the complexity of the regulatory network [19]. These studies highlight the diversity and importance of TFs in plant abiotic stress tolerance, thereby providing valuable gene resources for cold tolerance breeding. Beyond transcription factors, various other functional proteins also play indispensable roles in plant abiotic stress adaptation, among which selenium-binding proteins (SBPs) have attracted increasing attention due to their unique biological functions.

Selenium (Se) is one of the important essential trace elements for most microorganisms, animals, and humans [20]. SBPs are a class of selenium-containing proteins that are widely distributed, generally found and highly conserved in living species. It was first identified by Bansal et al. in mouse liver as a 56 kDa cytoplasmic protein capable of binding radioactive selenium; this protein was originally termed SBP56 [21], and later designated as SBP1 [22]. *SBP1* has anticancer action and is widely expressed in normal human tissues; its expression is significantly downregulated in malignant tissues, and dietary selenium supplementation decreases the risk of tumor formation [23]. Researchers initially found SBPs in the moss *Physcomitrella patens*, revealing that they may bind excess selenium within plants [24]. The similarity of SBPs between mammals (mouse, human) and plants (*Arabidopsis*) has been found to be high, exceeding 70%, and SBP1 has been identified as a new stress response regulator in plants [25]. Since the discovery of SBP in plants, researchers have been investigating their various roles and functions in plant physiology. Overexpression of *SBP1* increases cadmium tolerance in *Arabidopsis*, indicating that SBP1 may mediate a unique detoxification mechanism by directly binding metals to reduce heavy metal toxicity [26]. In *Arabidopsis*, transgenic plants with overexpression of *AtSBP1* demonstrated enhanced tolerance to sodium selenite, whereas transgenic plants with downregulated expression of *AtSBP1* were more sensitive [27]. SBPs also participate in plant defense against pathogens. Treatment of rice plants with rice blast fungus substantially increased *OsSBP* expression [28]. Overexpression of *OsSBP* promotes plant resistance to different pathogens, such as the rice blast fungus (*Pyricularia grisea*) and rice bacterial blight (*Xanthomonas oryzae pv. oryzae*) [29]. *LjSBP* is expressed in the *Arabidopsis* roots, cotyledons, and seed pods, and its expression levels are upregulated during plant growth and development. It may also engage in physiological processes like the redox status of target proteins and vesicle-Golgi transport [30]. *SBP* genes can integrate into regulatory networks to maintain cellular redox homeostasis and thereby regulate plant stress responses. *Chlorella selenium-binding protein domain 1* (*SBD1*), a homolog gene of *SBP1*, displays a lower ability to regulate oxidative stress in *sbd1* mutants relative to the wild type [31]. The *Arabidopsis SBP* gene family may contribute to mechanisms that detect redox imbalance [32]. Collectively, studies on *SBPs* in plants reveal their diverse roles in plant physiology.

This study revealed *LhSBP1* as a key gene that responds to cold stress in *Liriodendron* hybrids. Phenotypic observations and physiological parameter assessments demonstrated LhSBP1’s cold tolerance function. The overexpression of *LhSBP1* in transgenic *Arabidopsis* and ‘Nanlin 895’ poplar reduced ROS and MDA levels while enhancing antioxidant enzyme activity and soluble protein content under cold stress, consequently improving plant cold tolerance. This study provides a theoretical framework for further understanding LhSBP1’s complicated biological functions in regulating plant growth, development, and stress responses.

## 2. Results

### 2.1. Cloning, Bioinformatics Analysis, and Subcellular Localization of LhSBP1

**Figure 1:**
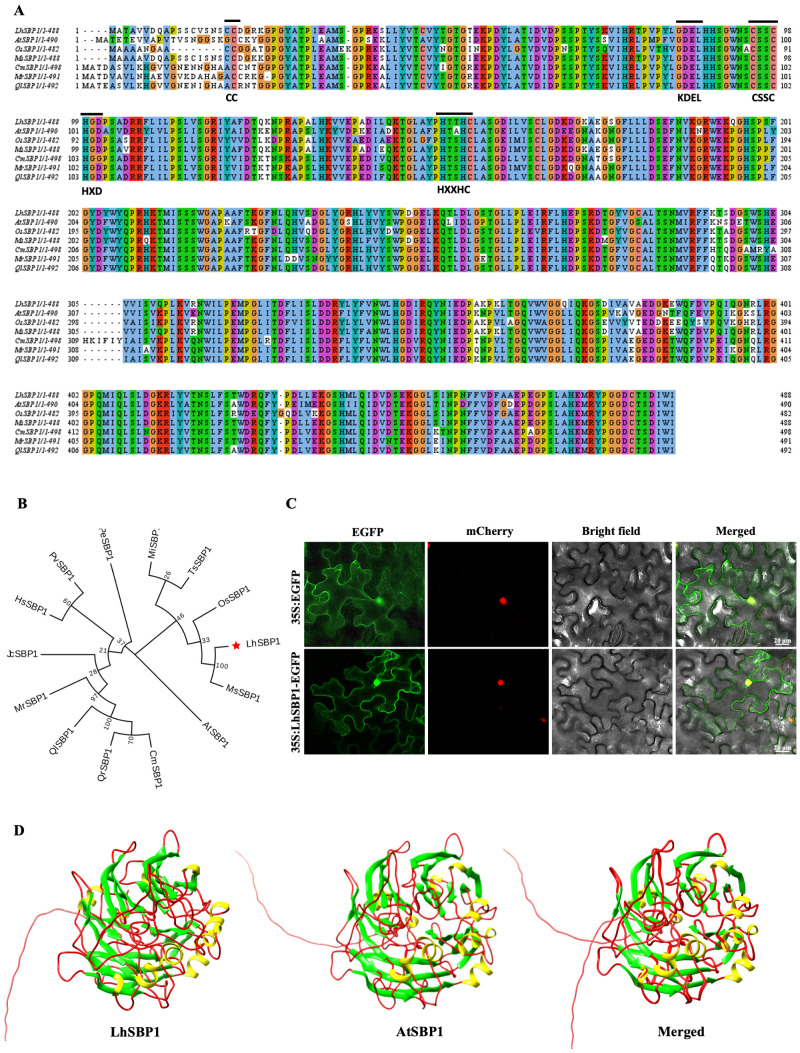
Sequence Analysis and Subcellular Localization of LhSBP1. Note: Species abbreviations: Lh, *Liriodendron* hybrid; At, *Arabidopsis thaliana*; Os, Oryza sativa; Ms, *Manglietiastrum sinicum*; Cm, *Cinnamomum micranthum*; Qr, *Quercus rubra*; Ql, *Quercus lyrata*; Ts, *Tetracentron sinense*; Mr, *Myrica rubra*; Hs, *Hibiscus syriacus*; Jc, *Jatropha curcas*; Pe, *Populus euphratica*; Mi, *Macadamia integrifolia*; Pv, *Pistacia vera*. (**A**) Multiple sequence alignment of SBP1 proteins from seven species (Lh, At, Os, Ms, Cm, Mr, Ql,), conserved motifs: CC, CSSC, HXD, HXXHC and KDEL; (**B**) Phylogenetic tree of SBP1 proteins from 14 species (Qr, Ql, Os, Mr, Hs, Jc, Pe, Ts, Mi, Cm, Lh, Ms, Pv, At). The red star highlights LhSBP1; (**C**) Subcellular localization of LhSBP1 in tobacco leaf cells. The 35S:LhSBP1-EGFP fusion protein was localized to the nucleus and cytoplasm; the 35S: EGFP empty vector served as a control, Scale bar = 20 um (**D**) Tertiary structures of LhSBP1 (left), AtSBP1 (middle), and their merged structure (right).

In this study, only one homologous gene of *Arabidopsis thaliana Selenium-Binding Protein 1* (*AtSBP1*) was identified in the *Liriodendron* hybrid genome (http://www.magnoliadb.com:7777/, accessed on 24 September 2024), with a sequence identity of up to 75.3%, and it was named *LhSBP1*. Based on the *Liriodendron* hybrid genome sequence, specific primers were designed. PCR amplification was performed using cDNA as a template, and a single target band was detected by agarose gel electrophoresis (Figure S1). Sequencing results showed that the full-length *LhSBP1* gene was 1467 bp, encoding 488 amino acids. Multiple sequence alignment of SBP1 proteins (Figure 1A) revealed high sequence similarity among these proteins. The core conserved motifs of SBP1 are mainly composed of cysteine- and histidine- related metal-binding and functional motifs. LhSBP1 includes five conserved motifs: CC, CSSC, HXD, HXXHC, and KDEL. SBP1 protein sequences from 14 species were downloaded from the NCBI database to construct a phylogenetic tree (Figure 1B), and the results indicated that LhSBP1 from *Liriodendron* hybrid has the closest genetic relationship with MsSBP1 from *Manglietiastrum sinicum*.

We performed subcellular localization of the LhSBP1 protein. 35S:*LhSBP1*-EGFP vector was transiently expressed in tobacco plants. Simultaneously, the empty 35S:EGFP vector was transformed into tobacco leaves as controls. Fluorescence distribution was examined using laser scanning confocal microscopy. Results showed that the fluorescence signal of the control was detectable throughout the tobacco cells, while the fluorescence signal of the experimental 35S:*LhSBP1*-EGFP was detectable in both the nucleus and cytoplasm (Figure 1C), indicating that the *LhSBP1* protein localizes to both the nucleus and cytoplasm.

Using the SWISS-MODEL database for homology modeling, the LhSBP1 protein’s tertiary structure was predicted to be irregularly coiled and stretched. Overlaying the tertiary structures of LhSBP1 and AtSBP1 with ChimeraX v1.7 software revealed a substantial similarity between the two proteins (Figure 1D). The cis-acting elements in the *LhSBP1* promoter sequence were predicted and analyzed using the PlantCARE database (Figure S2). The results showed that in addition to Abscisic Acid (ABA) signaling pathway response element, it contained regulatory elements closely associated with light response, defense, and stress; binding sites involved in drought induction; and elements participating in Methyl Jasmonate (MeJA) response.

### 2.2. Overexpression of LhSBP1 Enhances Cold Tolerance in Arabidopsis

**Figure 2:**
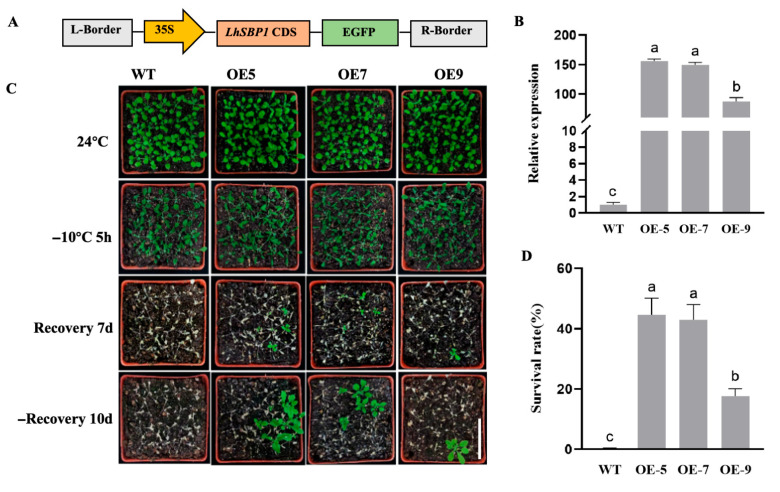
Construction and screening of *LhSBP1*-overexpressing transgenic *Arabidopsis*, and phenotypes and survival rates of *LhSBP1* transgenic lines OE-5, OE-7, and OE-9 under cold stress. (**A**) Schematic diagram of the overexpression vector; (**B**) Relative expression levels of the *LhSBP1* gene in *Arabidopsis* overexpression lines OE-5, OE-7, and OE-9; (**C**) Phenotypes of WT and OE *Arabidopsis* after recovery for 7 and 10 days following 5 h treatment at −10 °C. Scale bar = 5 cm; (**D**) Survival rates of WT and OE *Arabidopsis* after recovery for 10 days following 5 h treatment at −10 °C. Data represent the mean ± standard deviation (SD) from three independent replicates. Different lowercase letters above the bars indicate significant differences determined by Duncan’s Multiple Range Test (DMRT) at *p* < 0.05.

To investigate the effect of *LhSBP1* overexpression on *Arabidopsis* cold tolerance, an *LhSBP1* overexpression vector plasmid was constructed (Figure 2A). The plasmid was transformed into *Arabidopsis* via *Agrobacterium*-mediated transformation. Positive plants were obtained and subjected to Real-time quantitative PCR (RT-qPCR) analysis (Figure 2B), which shows that the overexpressing transgenic lines OE-5, OE-7, and OE-9 had significantly higher *LhSBP1* gene expression levels than wild-type (WT) plants. These three lines were selected for further investigation of *LhSBP1* in *Arabidopsis* cold tolerance; *Arabidopsis* seedlings (WT, OE-5, OE-7, and OE-9) that had been grown in soil for approximately three weeks and exhibiting similar growth vigor were used for subsequent experiments. The seedlings were subjected to cold treatment at −10 °C for 5 h, followed by recovery cultivation under normal growth conditions. Phenotypic observations were recorded at 7 and 10 days of recovery (Figure 2C), and survival rates were statistically analyzed after 10 days of recovery (Figure 2D). As shown in Figure 2C, both WT and transgenic lines exhibited severe stress after cold treatment, characterized by leaf vitrification. After 7 days of recovery culture, WT plants had essentially died and could not resume growth, whereas transgenic plants remained viable. After 10 days, it was evident that some rosette leaves of the *LhSBP1* transgenic plants were viable and green. OE-5 and OE-7 exhibited survival rates of up to 45%, as well as stronger cold tolerance than OE-9. As a result of the above findings, it is clear that overexpression of *LhSBP1* can improve *Arabidopsis* cold tolerance, and this is connected to the level of overexpression of *LhSBP1*, with higher expression levels resulting in stronger cold tolerance.

### 2.3. Effects of Cold Treatment on Physiological and Biochemical Characteristics of LhSBP1 Transgenic Arabidopsis

**Figure 3:**
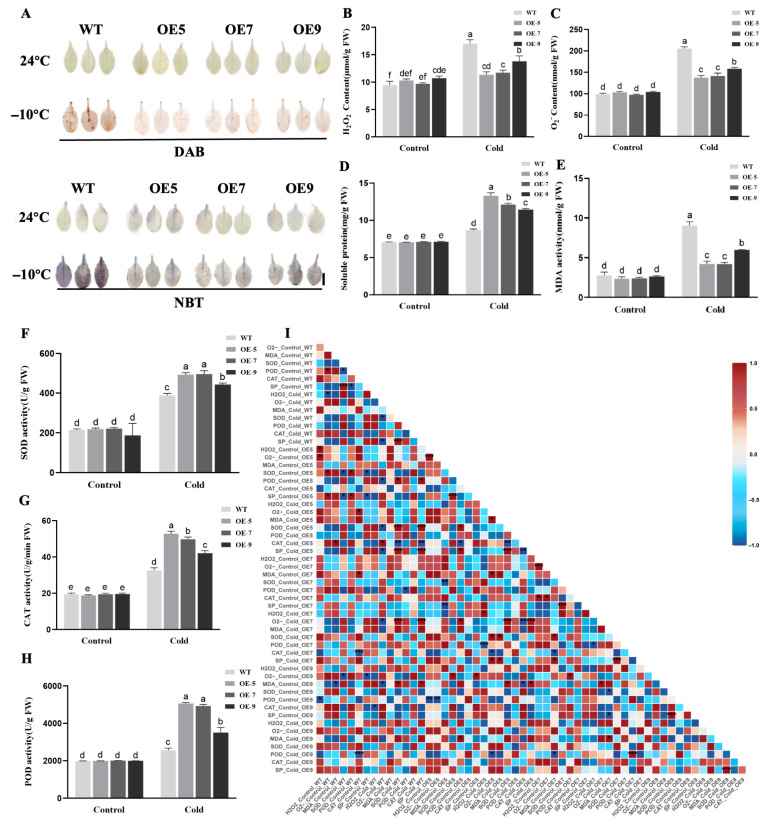
Physiological changes in WT and transgenic *Arabidopsis* lines OE-5, OE-7, and OE-9 under cold stress. (**A**) Histochemical staining of H_2_O_2_ (DAB) and O_2_^−^ (NBT) in Arabidopsis leaves. Scale bar = 1 cm; (**B**) H_2_O_2_ content; (**C**) O_2_^−^ content; (**D**) Soluble protein content; (**E**) MDA content; (**F**) SOD activity; (**G**) CAT activity; (**H**) POD activity; (**I**) Heat map of correlation analysis among the above physiological indicators. Data represent mean ± standard deviation (SD) from three replicates. Different lowercase letters above the bars indicate significant differences determined by Duncan’s Multiple Range Test (DMRT) at *p* < 0.05. The asterisk (*) above the bars shows significant differences (* *p* < 0.05, ** *p* < 0.01, *** *p* < 0.001).

To investigate the physiological mechanisms underlying *LhSBP1* overexpression lines after cold treatment, 3,3′-Diaminobenzidine (DAB) and Nitro Blue Tetrazolium (NBT) staining were performed on leaves of WT and OE *Arabidopsis* under cold stress (−10 °C, 5 h) (Figure 3A). Our results showed no significant difference in staining intensity between the two groups under normal growth conditions, with both showing minimal staining. Following cold treatment, WT leaves exhibited the deepest staining intensity, while the three transgenic lines (OE-5, OE-7, OE-9) showed lighter staining. The contents of hydrogen peroxide (H_2_O_2_) (Figure 3B) and superoxide anion (O_2_^−^) (Figure 3C) in *Arabidopsis* leaves were further detected. It was found that untreated WT and OE *Arabidopsis* plants exhibited similar levels. After cold treatment, the concentration of H_2_O_2_ and O_2_^−^ in both WT and OE *Arabidopsis* plants increased significantly. However, the rising levels of H_2_O_2_ and O_2_^−^ concentrations in OE lines were significantly lower than those in WT plants. The OE-5 and OE-7 lines exhibited the lowest O_2_^−^ content at just 0.67 and 0.69 times that of WT, respectively. This was significantly lower than the OE-9 line. These findings concurred with the histochemical staining observations. Correlation analysis further demonstrated a strong positive correlation between H_2_O_2_ and O_2_^−^ levels in OE-5 after cold treatment (Figure 3I), indicating consistent accumulation trends of reactive oxygen species (ROS) in transgenic lines under cold stress. As a result, it may be concluded that overexpression of *LhSBP1* can lower ROS generation in *Arabidopsis* under cold stress, hence mitigating the oxidative damage produced by cold stress.

Research revealed that under cold treatment, OE lines exhibited significantly higher soluble protein (SP) content compared to WT *Arabidopsis* (Figure 3D), with OE-5, OE-7, and OE-9 showing increases of 1.27, 1.28, and 1.14 times, respectively. Malondialdehyde (MDA) content of OE lines was significantly lower than WT, with OE-5 and OE-7 plants displaying MDA levels only 0.47 times that of WT (Figure 3E). These findings suggest that *LhSBP1* overexpression may enhance *Arabidopsis* cold tolerance by increasing soluble protein content and reducing membrane lipid peroxidation. After cold treatment, compared with WT, the activities of superoxide dismutase (SOD) (Figure 3F), catalase (CAT) (Figure 3G), and peroxidase (POD) (Figure 3H) in OE lines were significantly increased. Among the OE lines, OE-5 exhibited the highest SOD, CAT, and POD activities, reaching 1.27, 1.62, and 1.97 times that of WT, respectively. The SOD, CAT, and POD activities in OE-9 plants were lower than those in OE-5 and OE-7 plants, and showed a certain correlation with changes in ROS content in *Arabidopsis* under cold stress. For example, the O_2_^−^ content in OE-9 and OE-5 plants after cold treatment showed a strong negative correlation with POD activity. Additionally, in the low-temperature group of the overexpressing plants, the red positively correlated regions were all smaller than those in the WT group. The distribution of red and blue regions was more scattered. Especially, the negative correlation between antioxidant enzymes and oxidative damage indicators was more prominent, indicating that the association between the antioxidant system and damage indicators in the overexpressing lines was more stable (Figure 3I). These results show that overexpressing the *LhSBP1* gene effectively increases SOD, CAT, and POD activity to scavenge reactive oxygen species, enhancing *Arabidopsis* cold tolerance.

### 2.4. Overexpression of LhSBP1 Enhances Cold Tolerance in ‘Nanlin 895’ Poplar

**Figure 4:**
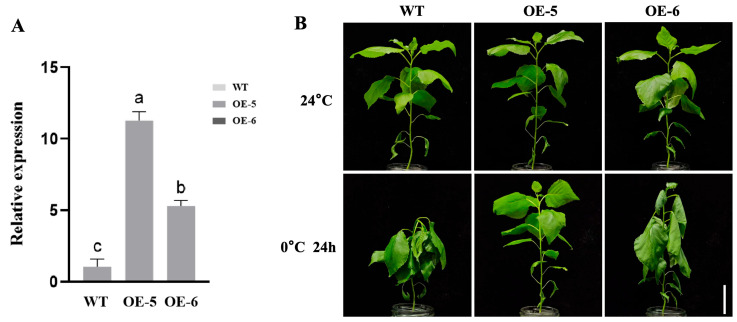
Screening of *LhSBP1*-overexpressing transgenic ‘Nanlin 895’ poplar lines and phenotypes in WT and *LhSBP1* OE lines under cold stress. (**A**) Relative expression levels of the *LhSBP1* gene in ‘Nanlin 895’ poplar overexpression lines OE-5 and OE-6; (**B**) Phenotypes of WT and *LhSBP1* OE lines after 24 h treatment at 0 °C. Scale bar = 5 cm. Data represent mean ± standard deviation (SD) from three replicate experiments. Different lowercase letters above the bars indicate significant differences determined by Duncan’s Multiple Range Test (DMRT) at *p* < 0.05.

**Figure 5:**
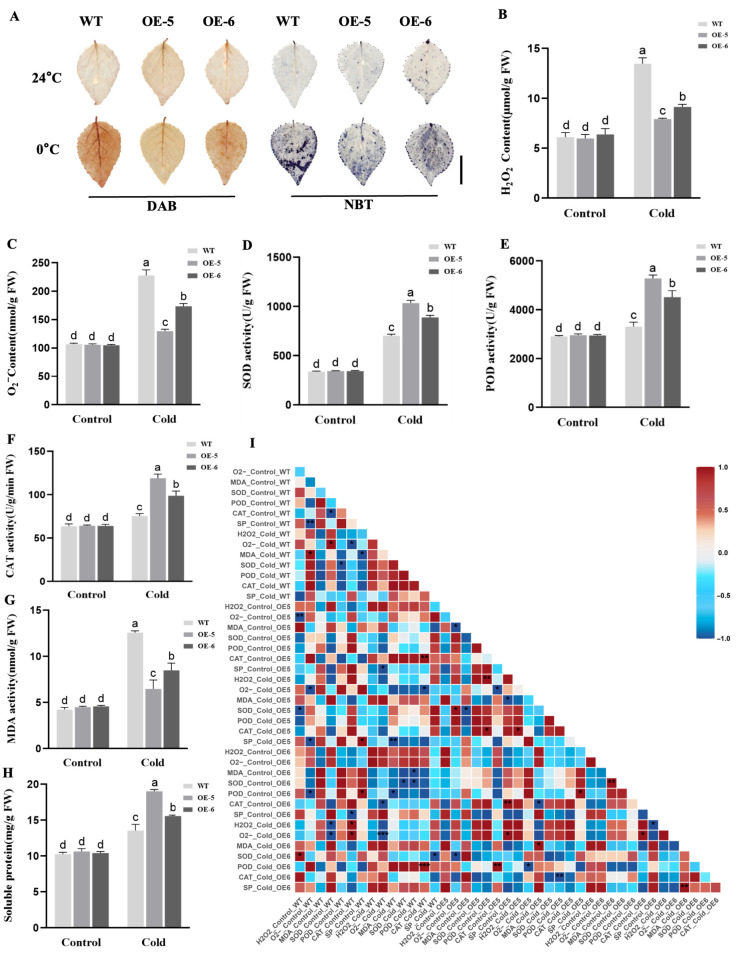
Physiological changes in WT and transgenic ‘Nanlin 895’ poplar lines OE-5 and OE-6 under cold stress (**A**) Histochemical staining of H_2_O_2_ (DAB) and O_2_^−^ (NBT) in Arabidopsis leaves. Scale bar = 3 cm; (**B**) H_2_O_2_ content; (**C**) O_2_^−^ content; (**D**) SOD activity; (**E**) POD activity; (**F**) CAT activity; (**G**) MDA content; (**H**) Soluble protein content; (**I**) Heat map of correlation analysis among the above physiological indicators. Data represent mean ± standard deviation (SD) from three replicates. Different lowercase letters above the bars indicate significant differences determined by Duncan’s Multiple Range Test (DMRT) at *p* < 0.05. The asterisk (*) above the bars shows significant differences (* *p* < 0.05, ** *p* < 0.01, *** *p* < 0.001).

*Agrobacterium rhizogenes* transformation of ‘Nanlin 895’ poplar resulted in transgenic seedlings, which went through the stages of co-cultivation, selection, regeneration, shoot elongation, and rooting. Ultimately, two transgenic lines, OE-5 and OE-6, with high expression levels were selected as the experimental materials (Figure 4A). To investigate the role of *LhSBP1* in cold tolerance of ‘Nanlin 895’ Poplar, three-month-old hydroponic seedlings of ‘Nanlin 895’ poplar (WT, OE-5, and OE-6) with identical growth rates were treated to 24 h of cold treatment at 0 °C, followed by phenotypic observation (Figure 4B). The results showed that the WT exhibited the most significant wilting after cold treatment. The OE-6 line wilted less than WT, whereas the OE-5 line wilted only a little at the apical bud. This suggests that the OE-5 line has higher cold tolerance than the OE-6 line, which is consistent with the level of *LhSBP1* overexpression. These findings indicate that *LhSBP1* overexpression enhances cold tolerance in ‘Nanlin 895’ poplar. Histochemical staining (DAB and NBT) was performed on the leaves of WT and OE lines under cold stress (0 °C, 24 h) (Figure 5A) to analyze the changes in ROS content in the leaves. The results showed that under normal growth conditions prior to cold treatment, there was no significant difference in staining intensity between the two groups of plants, and virtually no staining was observed. After cold treatment, the leaves of wild-type ‘Nanlin 895’ poplar showed the deepest staining, while the leaves of the two overexpression lines (OE-5 and OE-6) exhibited lighter staining. Further determination of H_2_O_2_ (Figure 5B) and O_2_^−^ (Figure 5C) contents in ‘Nanlin 895’ poplar leaves revealed that the contents were basically the same in WT and OE lines without cold treatment. As can be seen from Figure 5I, this triangular heatmap illustrates the correlations between ROS (including H_2_O_2_ and O_2_^−^), antioxidant enzymes (SOD, POD, and CAT), as well as MDA and soluble proteins, across different treatments (control/cold) and genotypes (WT, OE-5, and OE-6). The correlation between H_2_O_2_ and O_2_^−^ in WT and OE lines was weak under normal conditions. After cold treatment, the contents of H_2_O_2_ and O_2_^−^ in both WT and OE lines increased significantly, but the increment in OE lines was considerably lower than that in WT plants. The contents of H_2_O_2_ and O_2_^−^ in the OE-5 line were the lowest, only 0.59 and 0.57 times those of the WT, which is consistent with the histochemical staining, and the content change trends of H_2_O_2_ and O_2_^−^ were consistent. After cold treatment, the H_2_O_2_ and O_2_^−^ levels in OE-5 and OE-6 showed a strong positive correlation (Figure 5I). Therefore, overexpression of LhSBP1 can alleviate oxidative damage caused by cold stress in ‘Nanlin 895’ poplar.

The study analyzed antioxidant enzyme activities in WT and OE lines under cold treatment. Results indicated no significant differences in SOD (Figure 5D), POD (Figure 5E), and CAT (Figure 5F) activities between untreated WT and OE lines plants. Following cold treatment, the activities of SOD, CAT, and POD in both transgenic (OE) lines and WT were significantly increased. This increase in antioxidant enzyme activities correlated with phenotypic changes under cold stress and showed associations with ROS content. For example, there was a strong negative correlation between ROS (H_2_O_2_, O_2_^−^) and antioxidant enzymes (SOD, CAT) in OE-6. Additionally, soluble protein generally showed weak correlations with other oxidative indicators across all groups, whereas H_2_O_2_ and MDA consistently showed strong positive correlations, indicating that they are synchronous markers of oxidative damage (Figure 5I). Further measurements of soluble protein and MDA contents in WT and OE lines were conducted, and the results showed that after cold treatment, MDA content in OE lines was significantly elevated but remained markedly lower than in WT, averaging 0.6 times that of WT (Figure 5G). Soluble protein content in OE lines increased to 1.4 and 1.15 times that of WT (Figure 5H). Therefore, it can be concluded that overexpression of the *LhSBP1* gene effectively enhances the activities of SOD, CAT, and POD in ‘Nanlin 895’ poplar, thereby scavenging ROS, increasing soluble protein content, and reducing membrane lipid peroxidation, thereby improving the cold tolerance of ‘Nanlin 895’ poplar.

## 3. Discussion

As an evolutionarily conserved functional protein, selenium-binding protein 1 (SBP1) mainly exerts its functions in the plant’s response to abiotic stress through pathways such as oxidative stress regulation, metal detoxification, and signal molecule mediation [33]. In *Arabidopsis*, *SBPs* comprise three members: *SBP1*, *SBP2*, and *SBP3* [32]. This study identified only one homologous gene in *Liriodendron* hybrid that shares high similarity with *AtSBP1*, with an identity of 75.3%. By simultaneously analyzing SBP1 protein sequences from seven species, including *Liriodendron* hybrid, Arabidopsis, *Manglietiastrum sinicum*, and other species (Figure 1A), we found high similarity among SBP1 proteins across these seven species, which is consistent with previous findings that SBPs are highly conserved during evolution [27]. Furthermore, specific motifs have been identified as conserved features within SBP proteins. These include CC/CXXC, KDEL, CSSC, HXD, and HXXHC. These characteristic sequence motifs predominantly appear in archaea and bacteria, while being retained in plants and animals, with each motif playing distinct, crucial roles [25]. Studies have revealed that all SBPs in *Arabidopsis* are expressed in both the cytoplasm and nucleus of the protoplast system [34]. Subcellular localization analysis of LhSBP1 shows a similar expression pattern, whereas in wheat, SBP homologs are localized exclusively to the cytoplasm [35]. This study conducted cis-acting element prediction and analysis of the *LhSBP1* promoter sequence via the PlantCARE database, revealing that the promoter region of the *LhSBP1* gene contains multiple regulatory elements (Figure S2). These include elements closely associated with defense and stress regulation, as well as elements involved in MeJA response. Although no elements directly related to cold response were identified, recent studies have shown that MeJA also plays an essential role in plant defense against cold stress [36]. To verify the functional significance of these predicted cis-elements, we plan to generate truncated promoter-reporter gene constructs and carry out transient expression experiments in tobacco [37]. In the construction of transgenic plants, CaMV35S is the most widely used constitutive promoter. Its strong promoter activity in plants ensures the efficient expression of target genes [38,39]. However, the 35S promoter-induced constitutive expression fails to mimic the cold-inducible pattern of endogenous LhSBP1, and its non-physiological continuous driving may also impose metabolic burdens on plants or disrupt native regulatory networks [40,41,42]. To address these limitations, we will use the *Arabidopsis rd29A* promoter. This promoter maintains low basal expression under normal conditions but is activated by cold, and its stable inducibility in non-model plants has been confirmed [43]. Constructing rd29A::*LhSBP1* vectors will help accurately quantify LhSBP1’s functional contribution by comparing cold-resistant phenotypes between 35S- and rd29A-driven transgenic lines.

Cold stress can disrupt plant ROS regulation, change cell membrane fluidity, damage protein complexes, and reduce enzyme activity [44]. Enhancing antioxidant enzyme activity and osmotic regulator content constitutes a key strategy for plants to tolerate abiotic stress [45,46,47]. When ROS accumulate excessively under stress, they adversely affect cellular metabolism, disrupting normal cell functions, and causing oxidative damage [7]. Exogenous selenium compounds have been shown in studies to upregulate all three *SBP* genes in *Arabidopsis*. Additionally, *AtSBP1* responds to ROS and integrates into regulatory networks that maintain cellular redox homeostasis [32]. Studies on oxidative stress transcription in *Arabidopsis* also revealed *SBP1* upregulation in response to ROS [48]. In this study, following cold treatment, wild-type *Arabidopsis* and ‘Nanlin 895’ poplar accumulated more ROS than *LhSBP1* overexpression plants, causing irreversible cellular damage consistent with their phenotypic responses. MDA, a marker of lipid peroxidation and a key indicator of oxidative damage, is reduced in plants with improved growth [49]. Under cold stress, MDA accumulation was lower in *LhSBP1* overexpression *Arabidopsis* and ‘Nanlin 895’ poplar plants compared to wild-type plants, indicating reduced tissue damage. Accumulation of osmotic regulators, such as soluble carbohydrates and soluble proteins, helps lower osmotic pressure and stabilize cell membranes, thereby enhancing plant tolerance to cold stress [50]. This study found that overexpression of the *LhSBP1* gene increased soluble protein levels in *Arabidopsis* and in ‘Nanlin 895’ poplar under cold stress, thereby reducing cold-induced damage. SOD, POD, and CAT form an enzyme system that scavenges free radicals, removing excess ROS created within cells and therefore mitigating the oxidative damage induced by cold stress [51]. Under cold stress, *LhSBP1* overexpressing *Arabidopsis* and ‘Nanlin 895’ poplar had higher SOD, POD, and CAT activities than their respective wild-type. Thus, the *LhSBP1* gene likely improves cold tolerance by enhancing antioxidant enzyme activity and soluble protein content, thereby reducing excessive ROS accumulation and membrane lipid peroxidation, thereby regulating osmotic balance. Cold tolerance in *LhSBP1*-overexpressing ‘Nanlin 895’ poplar was more pronounced than in transgenic *Arabidopsis*. For instance, the O_2_^−^ content in the *Arabidopsis* OE-5 line was at least 0.67 times that of wild-type *Arabidopsis* (Figure 3C), while the O_2_^−^ content in the ‘Nanlin 895’ poplar OE-5 line was at least 0.57 times that of wild-type poplar (Figure 5C). This suggests that, during conservative evolution, this gene may also be undergoing optimization, yielding improved cold tolerance in woody plants.

Agalou et al. proposed that *AtSBP1* participates in a novel protein network, and confirmed via yeast two-hybrid system and pull-down assays that AtSBP1 interacts with 14 other proteins associated with vesicle transport, membrane synthesis, and redox regulation [52]. Further investigation is needed to determine whether LhSBP1 participates in the cold stress response of *Liriodendron* hybrids via protein interactions. A yeast library screen will be conducted to identify LhSBP1-interacting partners and explore downstream pathways that mediate its cold-tolerance activity. The complex protein interaction network of SBP1 may suggest new directions for cold tolerance interaction networks. *Liriodendron* hybrid is an intentionally engineered interspecific hybrid of *Liriodendron chinense* and *Liriodendron tulipifera.* It shows remarkable hybrid vigor, with straighter trunks, more ornamental leaves, and faster growth. It has become the preferred replacement species for forest restoration in southern China [53]. Cold stress not only impairs germplasm conservation in *Liriodendron* hybrids but also inhibits their northern spread. Thus, studying their biological responses to cold stress is critical. In this study, poplar was used as a model plant, therefore providing a solid reference framework for future research on the *LhSBP1*-transgenic *Liriodendron* hybrid.

## 4. Materials and Methods

### 4.1. Experimental Materials

Wild-type (WT) seed of *Arabidopsis thaliana* ecotype Columbia (Col-0) was maintained in the Laboratory of Biochemistry and Molecular Biology, Nanjing Forestry University, Nanjing, Jiangsu, China. ‘Nanlin 895’ poplar (*Populus deltoides × P. euramericana* cv. ‘Nanlin 895’) material was obtained from Nanjing Forestry University and subcultured in the same laboratory.

### 4.2. Total RNA Extraction from Liriodendron Hybrid, cDNA Synthesis, LhSBP1 Cloning, and RT-qPCR

Total RNA extraction from *Liriodendron* hybrid was performed using the FastPure Plant Total RNA Isolation Kit (RC401-01, Vazyme, Nanjing, China) [54]. cDNA synthesis was carried out with the SPARKscript II All-in-one RT SuperMix for qPCR (AG0305-A, SparkJade, Jinan, China) [55] and the synthesized cDNA was stored at −20 °C. Cloning primers were designed based on the full-length ORF of *LhSBP1*, and PCR amplification was conducted using the cDNA as a template. Real-time quantitative PCR (RT-qPCR) was performed using SYBR Green *Pro Taq* HS (AG11718, Accurate Biology, Changsha, China) [56] in Applied Biosystems QuantStudio^TM^ 1 Plus RT-qPCR machine (Thermo Fisher Scientific, Waltham, MA, USA). *Atactin* and *Pdactin* served as internal reference genes for *Arabidopsis* and ‘Nanlin 895’ poplar, respectively. Relative gene expression was calculated using the 2^−^^ΔΔCt^ method with three biological replicates [57]. Primer sequences are detailed in Table S1.

### 4.3. Bioinformatics Analysis of Liriodendron Hybrid LhSBP1 Gene

The AtSBP1 protein sequence from *Arabidopsis* was downloaded from the TAIR database. Homologous sequences in *Liriodendron* hybrid were identified using NCBI BLAST+ (E-value < 10^−5^) [58]. Representative plant SBP1 protein sequences were downloaded from the NCBI database. Using DNAMAN with default parameters, multiple sequence alignments were performed for SBP1 protein sequences from *Liriodendron* hybrid and other species [59]. A phylogenetic tree was constructed using the Neighbor-Joining (NJ) method in MEGA-X, with 1000 bootstrap replicates [60]. The online tool PlantCARE (http://bioinformatics.psb.ugent.be/webtools/plantcare/html/, accessed on 10 October 2024) was used to predict cis-acting elements in the promoter [61]. The Swiss-Model online software (https://swissmodel.ex-pasy.org/, accessed on 10 October 2024) was used to indicate the tertiary structure of the LhSBP1 protein and AtSBP1 protein, and ChimeraX v1.7 software was utilized for tertiary structural visualization analysis [62].

### 4.4. Subcellular Localization of LhSBP1 Protein

Construct the 35S: *LhSBP1*-EGFP fusion expression vector and transform it into *Agrobacterium* GV3101. Select 5–6-week-old *Nicotiana tabacum* (*N. tabacum*) plants for transient transformation. *Agrobacterium* containing the recombinant plasmid was injected into tobacco leaves. After 1 day of dark incubation, plants were transferred to normal light conditions for 2 days [63]. Fluorescence imaging was performed using a Leica TCS SP8 confocal laser scanning microscope (Leica Microsystems, Wetzlar, Germany).

### 4.5. Arabidopsis Genetic Transformation and Positive Plant Screening Method

Wild-type *Arabidopsis* as transformed with the recombinant plasmid 35S:*LhSBP1*-EGFP using the *Agrobacterium*-mediated inflorescence-dipping method [64] to obtain T0 transgenic plants. After cultivation until seed production, T1 seeds were screened on 1/2 MS solid medium containing 50 mg·L^−1^ kanamycin, and positive seedlings were transferred to soil. When T1 plants developed 8–10 rosette leaves, DNA was extracted using the CTAB method [65] and PCR was performed to identify positive plants. RNA was extracted from T1 plant leaves, reverse transcribed into cDNA, and subjected to RT-qPCR amplification for strain selection. T2 seeds were collected from the selected T1 lines and re-screened on kanamycin-containing 1/2 MS medium to confirm the heritability of the target gene. Positive T2 seedlings were transplanted to soil and grown to maturity, and their *LhSBP1* expression stability was verified by RT-qPCR again. Finally, T3 seeds were harvested, and homozygous transgenic lines (T3) were obtained through continuous kanamycin screening and expression verification. These T3-homozygous lines, with stable inheritance and consistent *LhSBP1* expression, were used in subsequent cold-tolerance experiments to ensure the reliability and reproducibility of the results [66].

### 4.6. Genetic Transformation and Positive Plant Screening Method for ‘Nanlin 895’ Poplar

Cut the apical buds from 4 to 6-week-old ‘Nanlin 895’ poplar tissue culture seedlings and inoculate them onto *Agrobacterium* colonies. Transgenic plants were obtained through co-culture, selection, regeneration, shoot elongation, and rooting culture steps [67]. By extracting RNA from ‘Nanlin 895’ poplar leaves, reverse transcribing, and amplifying by RT-qPCR to confirm overexpression lines.

### 4.7. Measurement of Physiological and Biochemical Indicators Under Cold Stress

DAB and NBT staining: Prepare 1 mg/mL DAB and 0.2% NBT solutions. Immerse the plant leaf in the staining solution and incubate at room temperature (25 ± 2 °C) in the dark for 12 h. Perform gradient decolorization by treating with 75% ethanol solution in an 85 °C constant temperature water bath for 1 h [68].

Using Hydrogen Peroxide (H_2_O_2_) Content Assay Kit (AKAO009M, BoxBio, Beijing, China) to determine the content of H_2_O_2_ [69], and O_2_^−^ content was determined by Superoxide Anion Content Assay Kit (AKAO008M, BoxBio, Beijing, China) [70], SOD activity was determined using the Superoxide Dismutase (SOD) Activity Assay Kit (AKAO001M-50S, BoxBio, Beijing, China) [71]. In contrast, POD activity and CAT activity were measured with the Peroxidase Activity Assay Kit (AKAO005M, BoxBio, Beijing, China) [72] and Catalase Activity Assay Kit (AKAO003-2M, BoxBio, Beijing, China), respectively. MDA content and soluble sugar content were assayed using the Malondialdehyde Content Assay Kit (AKFA013M, BoxBio, Beijing, China) [73] and Plant Soluble Sugar Content Assay Kit (AKPL008M, BoxBio, Beijing, China) [74], respectively.

### 4.8. Data Analysis

Data for each variable underwent analysis of variance. Duncan’s Multiple Range Test (DMRT) was used to determine the significance of differences between treatment groups at a significance level of *p* < 0.05 [75]. GraphPad Prism 9 software was employed for graphical presentation. The values presented represent the mean of three independent experiments.

## 5. Conclusions

**Figure 6:**
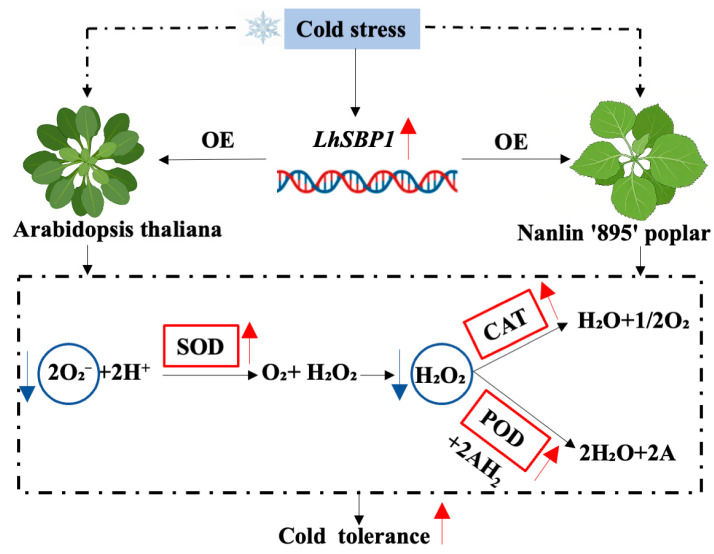
Schematic diagram of the *LhSBP1*-mediated cold stress tolerance pathway via regulating ROS metabolism in plants.

This study cloned the *LhSBP1* gene from the *Liriodendron* hybrid genome and transformed it into wild-type *Arabidopsis* and ‘Nanlin 895’ poplar. Under cold stress, transgenic *Arabidopsis* and ‘Nanlin 895’ poplar overexpressing *LhSBP1* maintained cellular ROS homeostasis. The activities of antioxidant enzymes (SOD, CAT, POD) and the content of soluble protein were significantly increased, while the contents of H_2_O_2_, O_2_^−^, and MDA were significantly lower. This alleviated oxidative damage and enhanced the cold tolerance of the transgenic plants (Figure 6). In summary, this study reveals that the LhSBP1 protein plays a role in ensuring cold tolerance, providing a theoretical basis for developing cold-tolerant *Liriodendron* hybrid varieties through transgenic technology. It also provides new insights into the northern development of *Liriodendron* hybrid cultivation.

## Data Availability

The original contributions presented in this study are included in the article/Supplementary Materials. Further inquiries can be directed to the corresponding author.

## Supplementary Materials

The following supporting information can be downloaded at: https://www.mdpi.com/article/10.3390/plants15020196/s1. Figure S1: Amplified bands of the *LhSBP1* gene; Figure S2: Analysis of cis-acting elements in the *LhSBP1* promoter; Table S1: Sequences of primers used in this study.
