# Supplementary material for: LhSBP1 Gene of Liriodendron Hybrid Enhances the Cold Resistance of Plants by Regulating ROS Metabolism
Source: Plants (Basel). 2026 Jan 8;15(2):196. doi: 10.3390/plants15020196 (PMC12845277; doi:10.3390/plants15020196)
Supplement: Supplementary file 1 [file plants-15-00196-s001.zip › Figure S1.pdf]

Figure S1

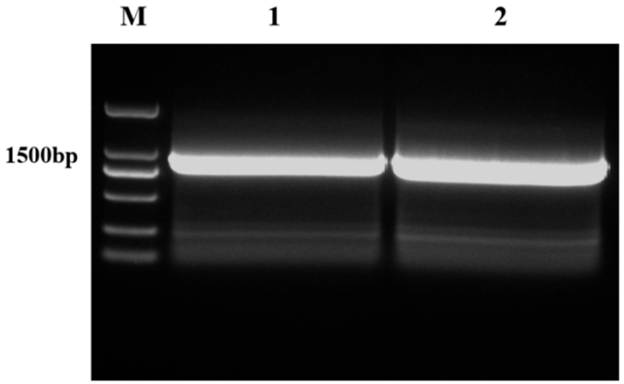

Figure S1. Amplified bands of the *LhSBP1* gene (Lanes 1 and 2: Amplified bands of *LhSBP1*; M: 2000bp DNA Marker)
