# Supplementary figures and images for: LhSBP1 Gene of Liriodendron Hybrid Enhances the Cold Resistance of Plants by Regulating ROS Metabolism
Source: Plants (Basel). 2026 Jan 8;15(2):196. doi: 10.3390/plants15020196 (PMC12845277; doi:10.3390/plants15020196)

Figure S2

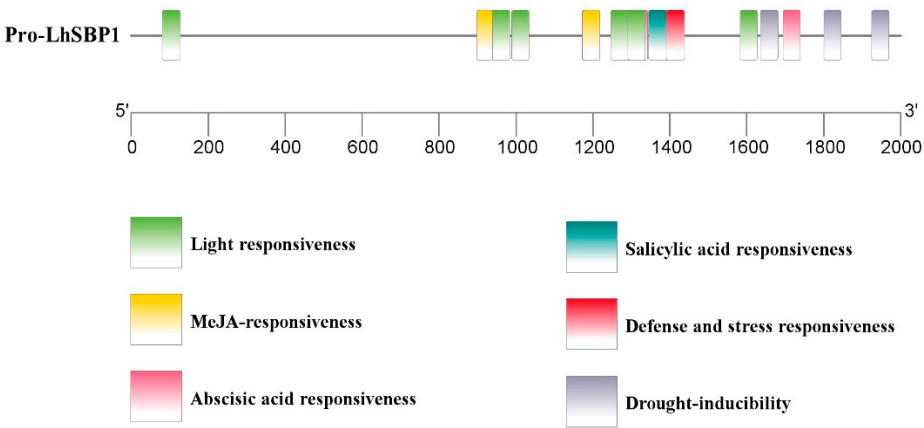

**Figure S2.** Analysis of cis-acting elements in the *LhSBP1* promoter

Supplement: Supplementary file 1 [file plants-15-00196-s001.zip › Figure S2.pdf]
